# Femoral Neck Fracture with Misdiagnosis of Osteonecrosis of the Femoral Head: A Two-Case Report

**DOI:** 10.3390/medicina60071063

**Published:** 2024-06-27

**Authors:** Ting-Hsien Kwan, Chen-Hao Chiang, Wei-Hsing Chih, Cheng-Ming Chou

**Affiliations:** Department of Orthopaedics, Chia-Yi Christian Hospital, Chiayi 600, Taiwan; med.madness923@gmail.com (T.-H.K.); chiangabaca@gmail.com (C.-H.C.); chihws@gmail.com (W.-H.C.)

**Keywords:** spontaneous femoral neck fracture, osteonecrosis, femoral head, total hip arthroplasty, misdiagnosis

## Abstract

We report two rare cases of femoral neck fracture resulting from osteonecrosis of the femoral head (ONFH) that was undiagnosed at the patients’ initial visits. The patient in the first case had sequential bilateral displaced femoral neck fractures. Because no osteonecrosis of the femoral head was visible on X-ray film and the data of liver function tests were normal, ONFH was not diagnosed. In addition, because the patient was a 55-year-old man with normal everyday functioning, closed reduction with cannulated screws was performed at both visits. Nine months later, he came to our outpatient department with bilateral hip pain; X-rays revealed nonunion and implant failure at both hips. The patient subsequently underwent bilateral total hip arthroplasty (THA) and had a satisfactory outcome at his 4-year follow-up. The patient in the second case had a left displaced femoral neck fracture after trivial trauma two months prior. ONFH was not diagnosed upon examination of X-ray findings. The patient was 52 years old with liver cirrhosis and had bipolar hemiarthroplasty performed because of a chronic displaced fracture and poor general condition. After 2 years, she began to have right hip pain. X-rays revealed massive necrosis and sclerosis of the femoral head. Computed tomography scans for ONFH staging revealed impending fracture lines at the subcapital site of the patient’s previous left femoral neck fracture. Right THA was then performed, and the outcome was satisfactory.

## 1. Introduction

Osteonecrosis of the femoral head (ONFH) is common in young to middle-aged patients. The disease presents itself with persistent hip pain and is diagnosed using X-ray or MRI. In rare cases, ONFH is associated with spontaneous fracture of the femoral neck [1,2].

When it comes to femoral neck fracture in young adults, surgical management with osteosynthesis is recommended to achieve an anatomic reduction in the fracture to preserve the blood supply and effectively prevent ONFH, and to provide a stable fixation while preserving bone stock to achieve union. Hence, compressive screws are suggested in Garden type I and II femoral neck fracture, whereas a dynamic hip screw is suggested in Garden type III and IV femoral neck fracture [3].

If ONFH is not identified at the fracture presentation, osteosynthesis with internal fixation may be performed in accordance with the above-stated principle. In this kind of scenario, osteosynthesis is doomed to fail because of the necrotic femoral head bone quality, and a secondary procedure for arthroplasty must be performed, resulting in a longer, more difficult recovery. Moreover, misdiagnosis of ONFH may lead to litigation. 

In this report, we present two rare cases of femoral neck fracture with misdiagnosis of ONFH.

## 2. Detailed Case Descriptions

**Figure 1:**
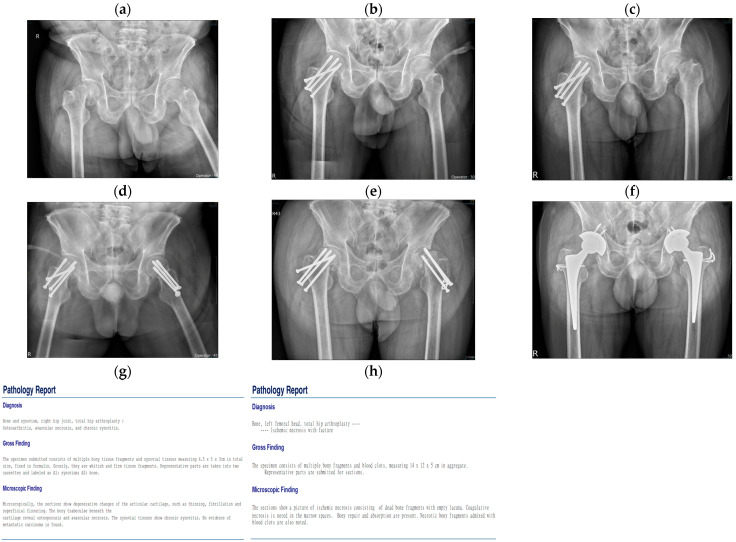
Case 1: A fifty-five-year-old man with a history of hypertension and a drinking history of 30 years. (**a**) Right femoral neck subcapital fracture without gross ONFH. (**b**) Internal fixation with cannulated screws for the right femoral neck fracture. (**c**) Left femoral neck subcapital fracture without gross ONFH. (**d**) Internal fixation with cannulated screws for the left femoral neck fracture. (**e**) Bilateral hips nonunion and implant failure. (**f**) Sequential bilateral total hip arthroplasty (THA). (**g**,**h**) Pathology reports of bilateral avascular necrosis at both femoral heads.

This case report study was approved by our institution’s review board. The patient in the first case was a 55-year-old man with a history of hypertension. The patient had a history of alcohol consumption, drinking one cup of 22% alcohol every day for 30 years. He visited our outpatient department on 7 May 2018. His chief complaint was a falling accident one week prior with minor right hip trauma. The patient experienced mild right hip pain that became intolerable over the next 2 days. X-ray film revealed a right femoral neck subcapital fracture with displacement (Figure 1a). The lab data for GOT, GPT, INR, and platelets were all within normal limits. Because no osteonecrosis of the femoral head was noted in the X-ray film, and the patient had led a normal life prior to the fracture, closed reduction and internal fixation with cannulated screws were performed (Figure 1b). He was discharged without any incident. Five weeks later, he came to our outpatient department complaining of severe left hip pain. X-rays revealed no left hip trauma; his left hip was severely strained due to overcompensating for a previous right hip condition. X-rays revealed a left femoral neck subcapital fracture without gross ONFH, a pattern identical to that observed in his previously injured right hip (Figure 1c). Closed reduction and internal fixation using cannulated screws were then performed (Figure 1d), and he was discharged without any incident. He did not follow up at our outpatient department until 9 months after the initial procedure when he came to our clinic complaining of bilateral hip pain. X-rays revealed bilateral nonunion and implant failure in both hips (Figure 1e). He then received sequential bilateral THA (Figure 1f). Pathology reports indicated bilateral avascular necrosis at both femoral heads. The patient recovered well in both hips when followed up after four years.

**Figure 2:**
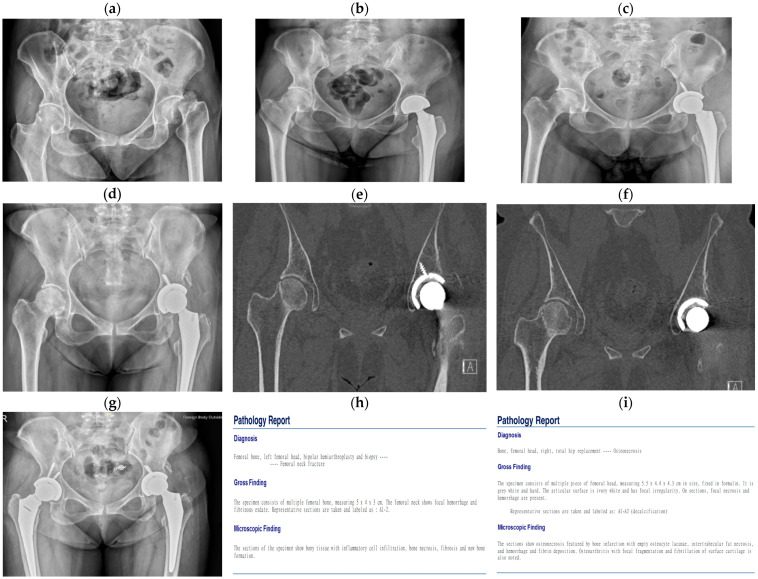
Case 2: A fifty-two-year-old woman with a history of liver cirrhosis and a drinking history of 30 years. (**a**) Left femoral neck subcapital fracture with displacement. (**b**) Left hip bipolar hemiarthroplasty for the left femoral neck fracture. (**c**) Left THA was performed after habitual prosthesis dislocation due to acetabular dysplasia and bone loss at the dislocation site. (**d**) X-ray showing ONFH over a large area on the right side. (**e**,**f**) CT scan in preparation for ONFH staging revealing an impending fracture line in the subcapital area at the same site as the previous left femoral neck fracture. (**g**) Right THA for right ONFH. (**h**,**i**) Pathology reports of bilateral avascular necrosis at both femoral heads.

The patient in the second case was a 52-year-old woman with a history of liver cirrhosis. She had a history of drinking two cups of alcohol every day for 30 years. She visited our outpatient department for left hip pain on 20 May 2021 after a minor falling accident 2 months prior. X-rays revealed a left femoral neck subcapital fracture with displacement (Figure 2a). Due to the chronic nature of the fracture and the patient’s limited mobility caused by her liver cirrhosis, left hip bipolar hemiarthroplasty (Figure 2b) was performed. After surgery, she experienced habitual prosthesis dislocation due to acetabular dysplasia and bone loss at the dislocation site. Treatment was changed to THA (Figure 2c) and the patient had a satisfactory outcome. After 2 years, she revisited our outpatient department, complaining of right hip pain over the previous 2 months. X-rays revealed right ONFH over a large area (Figure 2d). A CT scan was conducted for ONFH staging and showed an impending fracture line in the subcapital area at the same site as her previous left femoral neck fracture (Figure 2e,f). Right THA (Figure 2g) was performed, and pathology reports indicated bilateral avascular necrosis at both femoral heads. After follow-up, she had a smooth postoperative recovery.

To summarize our two cases, a case summary and comparison table are listed below in Table 1.

## 3. Discussion

Although the subchondral area is the most common site of bone fracture and collapse in cases of ONFH, there have also been sporadic case reports of ONFH resulting in spontaneous femoral neck fracture [1,2,4,5,6,7,8,9,10,11,12,13,14,15,16,17]. We reviewed all the reports listed in Table 2.

Several conclusions can be drawn from these cases. (1) Femoral neck fracture with ONFH occurs entirely at the subcapital sites. Additionally, the displaced fracture pattern revealed by plain X-ray film is nearly identical in every case. Compared with that of ordinary displaced femoral neck fractures, the fracture site in cases of ONFH is prominently located in the capital areas, the fracture line is straighter, and the fracture edge is smoother. (2) Because ONFH rarely progresses to a spontaneous femoral neck fracture, it is seldom diagnosed. (3) The epidemiology of femoral neck fractures after ONFH is nearly identical to ordinary ONFH, such as age, sex, and other risk factors. (4) All spontaneous femoral neck fractures after ONFH occur without prior trauma or with minor trauma history, suggesting that the actual incidence of spontaneous femoral neck fractures after ONFH may be more widespread than medical professionals realize. (5) In addition to personal history and X-ray film, a study using MRI or scintigraphy can confirm a diagnosis of ONFH. (6) The most common surgical procedures for treating spontaneous femoral neck fractures are bipolar hemiarthroplasty and THA. However, other surgical options are available, namely femoral head resurfacing [5], bone grafts [7], pinning or sliding hip screws [9,10], and core decompression [12]. 

Corticosteroid use, excessive alcohol consumption, trauma, blood coagulation disorders, hemoglobinopathy, autoimmune diseases, HIV [11], and smoking are highly correlated with ONFH risk [18]. These factors should arouse suspicion of ONFH in medical professionals treating a femoral neck fracture without obvious prior trauma. An accurate diagnosis of ONFH is critical to avoiding unnecessary surgical procedures; moreover, an accurate diagnosis of ONFH may enable preventive treatment of contralateral hip conditions before the conditions progress to spontaneous femoral neck fractures.

Furthermore, past studies revealed that ONFH and osteoporosis share common clinical and pathophysiological features, and ONFH is associated with low bone mineral density. One of these studies showed that fractural stages of ONFH were associated with a 5-fold risk of osteoporosis [19]. In one study, radiofrequency echographic multi-spectrometry (REMS) was developed as a practical ultrasound technique to evaluate osteoporosis, hip fracture risk, and occult hip fractures, which showed a correlation between osteoporosis and hip fracture risk [20]. One limitation of our case report is that there is no osteoporosis study of the hips before surgical management due to its presentation as fracture patterns at our clinic.

Previous studies have revealed that the pathological femoral neck fracture resulting from ONFH usually occurs at the subcapital area between the interface of necrotic and reparative bone [21,22,23,24]. When the fracture is without displacement, the femoral head may present as normal, and X-ray film alone is not sensitive enough to enable a conclusive diagnosis of ONFH [4]; however, if the fracture is displaced, as it often is, X-ray film is sufficient to suggest the presence of ONFH. Furthermore, the displaced fracture pattern was nearly identical in all the research cases discussed in this study. Specifically, in cases of ONFH, fractures are located close to the femoral head, have straight fracture lines, and have smooth fracture edges. The two patients treated in this case report exhibited displaced fracture patterns. In summary, the research we reviewed and the cases we treated suggest that it is reasonable to suspect ONFH-induced fractures if we are more familiar with this pathological situation, even when they are not readily visible on X-ray film.

Once ONFH is suspected from personal history or X-ray film, further studies may be warranted. Our research suggests that MRI can reveal a low-signal intensity band (representing the necrotic–viable bone interface) in T1-weighted images and a double-density line (representing hypervascular granulation tissue at the necrotic–viable bone interface) in T2-weighted images [4,25]. Due to a local inflammatory response from fracture, scintigraphy showed markedly increased radionuclide uptake, rendering it less helpful in identifying ONFH [4,16]. However, Yoon et al. made an ONFH diagnosis using pinhole bone scintigraphy in a case where plain film and MRI findings indicated no ONFH. In the second patient we treated, due to misdiagnosis, we had only a right hip CT scan showing massive ONFH and impending subcapital fracture. This CT scan clearly depicted the left subcapital condition before the spontaneous fracture occurred.

The most common treatment for spontaneous femoral neck fracture following ONFH is arthroplasty, either bipolar hemiarthroplasty or THA. The first patient we treated in this study received bilateral THA after the failure of cannulated screws. The second patient we treated had left bipolar fractures treated as regular femoral neck fractures. Treatment for her condition was changed to THA after frequent dislocation from acetabular dysplasia and bone loss due to bony impact resulting from her habitual dislocation at the site. The patient had ONFH of Ficat’s stage III on the right side with an impending fracture line at the subcapital site, and we performed right THA per our standard practice in such cases.

## 4. Conclusions

Misdiagnosis of ONFH at a femoral neck fracture may cause surgeons to conduct incorrect surgical strategies that require a second arthroplasty when the initial procedure fails. Moreover, suspicion of ONFH is warranted in cases of subcapital fractures in younger patients with minor trauma. In cases where patients have risk factors for ONFH, we suggest further study using MRI or scintigraphy to diagnose ONFH with greater certainty. Arthroplasty is the standard treatment for spontaneous femoral neck fracture with ONFH.

## Figures and Tables

**Table 1:** Case summary and comparison.

Case	Age	Gender	Underlying	Risk Factors	Fracture Site	Trauma History	Diagnostic Tool	Surgical Treatment
			Disease	Alcoholic Steroid Idiopathic				
No. 1	55	Male	Hypertension	Alcoholic	Subcapital	No	X-ray	1. Cannulated screws 2. THA
No. 2	52	Female	Liver cirrhosis	Alcoholic	Subcapital	No	X-ray CT	1. Bipolar 2. THA

THA: total hip arthroplasty.

**Table 2:** Reviews of spontaneous femoral neck fractures after ONFH.

Study	Case No.	Age (Mean)	Gender (M/F)	Risk Factors	Fracture Site	Trauma History	Diagnostic Tool	Treatment
				Alcoholic (A) Steroid (S) Idiopathic (I)				
Usui et al. Japan, 1996 [5]	4	43	0/4	A: 0 (0%) S: 4 (100%) I: 0 (0%)	Subcapital	No	X-ray	Resurfacing
Kim and Kim Korea, 2000 [4]	2	59	2/0	A: 1(50%) S: 1(50%) I: 0 (0%)	Subcapital	No	MRI Bone scan	Bipolar
Min et al. Korea, 2001 [2]	10	51.9	9/1	A: 8 (80%) S: 2 (20%) I: 0 (0%)	Subcapital	No	MRI	THA
Loddenkemper et al. Germany, 2002 [6]	1	47	0/1	A: 0 (0%) S: 1 (100%) I: 0 (0%)	Subcapital	No	X-ray	THA
Yoon et al. Korea, 2004 [7]	1	45	1/0	A: 0 (0%) S: 0 (0%) I: 1 (100%)	Subcapital	No	MRI Bone scan	Bone graft
Kamiya et al. Japan, 2004 [8]	1	60	0/1	A: 0 (0%) S: 1 (100%) I: 0 (0%)	Subcapital	No	MRI	Bipolar
Lee and Suh Korea, 2005 [9]	1	47	1/0	A: 1 (100%) S: 0 (0%) I: 0 (0%)	Subcapital	No	MRI	THA Pinning
Zuckerman et al. USA, 2006 [10]	1	46	0/1	A: 0 (0%) S: 1 (100%) I: 0 (0%)	Subcapital	No	MRI	Sliding hip screws
Tompkins et al. USA, 2010 [11]	3	48	1/2	A: 0 (0%) S: 1 (33%) I: 3 (67%)	Subcapital	No	X-ray	THA
Chang et al. Korea, 2010 [12]	1	23	1/0	A: 0 (0%) S: 1 (100%) I: 0 (0%)	Subcapital	No	MRI CT scan	Decompression
Vaishya India, 2012 [13]	1	47	1/0	A: 0 (0%) S: 0 (0%) I: 1 (100%)	Subcapital	No	CT scan	THA
Fukui et al. Japan, 2015 [14]	1	60	1/0	A: 0 (0%) S: 1 (100%) I: 0 (0%)	Subcapital	No	MRI	Bipolar
Kumar et al. India, 2016 [15]	1	42	1/0	A: 1 (100%) S: 0 (0%) I: 0 (0%)	Subcapital	Squatting	MRI	THA
Shah et al. India, 2019 [16]	1	28	1/0	A: 1 (100%) S: 0 (0%) I: 0 (0%)	Subcapital	Squatting	MRI	THA
Arora et al. India, 2021 [1]	12	32	9/1	A: 3 (30%) S: 7 (70%) I: 3 (30%)	Subcapital	No	CT scan or MRI	THA
Yen et al. Taiwan, 2021 [17]	1	30	1/0	1 (100%) S: 0 (0%) I: 0 (0%)	Subcapital	No	MRI	THA
This study Taiwan, 2023	2	53	1/1	2 (100%) S: 0 (0%) I: 0 (0%)	Subcapital	Minor	X-ray CT scan	THA

## Data Availability

The original contributions presented in this study are included in the article, further inquiries can be directed to the corresponding author.
